# Supplementary material for: MRI risk factors for development of avascular necrosis after closed reduction of developmental dysplasia of the hip: Predictive value of contrast-enhanced MRI
Source: PLoS One. 2021 Mar 18;16(3):e0248701. doi: 10.1371/journal.pone.0248701 (PMC7971487; doi:10.1371/journal.pone.0248701)
Supplement: Attachment — Submitted filename: Response to reviewers.docx [file pone.0248701.s001.docx]

Response to Reviewers

Reviewer #1: The subject of this study is interesting and relevant to paediatric orthopaedic surgeons. There is currently no "gold-standard" imaging that can safely predict the development of AVN of the femoral epiphysis following closed reduction for DDH. This study suggests an important first step in developing a gold standard. A few suggestions on improving the manuscript further follow.

1. Firstly, it would be interesting to add some information on the safety of the process. Have there been any reactions or side effects related to the administration of the contrast?

- Thank you for your comments. There have been no adverse reaction related to the gadolinium contrast materials in this study population.

2. Second, please can you elaborate further on why infants had to be sedated for the MRI? Does the hip spica cast not provide sufficient immobility for the MRI to be undertaken.

- Regarding sedation issue, it is related to limited MRI resources in our hospital. It is very difficult to adjust scanning schedule soon after mean or immediate after closed reduction. Therefore it is our routine practice to scanning MRI after sedation in children younger than 8-year-old.

3. The Authors have done a great job in designing the study as best as possible, considering this was a single-centre trial with relatively small numbers and limited follow-up. The weaknesses of the study are recognised and adequately discussed in the Discussion section.

- Thank you for your comments.

Some further discussion of the following points would be helpful for the reader:

4. The confounding factors (previous treatment with harness/brace, traction and adductor tenotomy) were discussed and their predictive value calculated. However, the numbers were perhaps too small to provide enough power for these calculations. Could the Authors comment on this issue? Would it not be more appropriate to just focus on the predictive value of MRI?
Thank you for your comments. There have been no adverse reaction related to the gadolinium contrast materials in this study population.

- Thank you for your comments. We revised the manuscript focused on MRI risk factors.
- We revised manuscript title as well as results according to your comments.

5. How well were the 3 patterns of contrast enhancement distinguishable? How easy was to define the 80% cut-off point?

- We qualitatively evaluated femoral head enhancement pattern. We divided the femoral head into four quadrants. If the non-enhancing areas are greater than 3/4 (75%, it was rounded off to 80%), it was considered global decreased enhancement. We revised this description as it was.

6. Is the abduction angle not affected by the position of the infant in the scanner? For example a more flexed or extended position of the pelvis resulting from the position of the infant in the spica and in the scanner would surely affect the abduction angle?

- It is a wonderful question. Hip abduction angle measure on axial images can be affected by hip flexion state. We added this issue in the discussion.

7. I am impressed by the consistency achieved in the usage of the Salter criteria for AVN. These criteria would probably cover the more severe AVN's. However, the lateral AVN causing tilting of the femoral epiphysis and coxa valga may not be evident for several years. It is claimed that the Salter criteria can predict Kalamchi classification at age 10 or older. Could the Authors expand on how solid this evidence is and comment on this issue further?

- I agree you opinion. We also reviewed all postoperative radiographs in this patients.

If there were disagreement between two readers, final interpretation was determined using a combination of Salter criteria and Kalamchi classificaxtion in consensus.

8. I fully agree that these results require to be confirmed in multi-centre settings with longer follow-up. This paper is a positive step in this direction.
The paper is well written and the text flows in a logical sequence. The illustrations are useful and appropriate.

- Thank you for your comments.

Reviewer #2

This is important work and although the reference standard test is somewhat limited because it is defined as “AVN determined by Salter’s criteria at only 12 months”, the study shows that there is indeed an important value of the test. However, despite of its limitations, it is robust enough and there was supportive data on its reliability. (The key issue in interpreting the results will be to consider scenarios of varying reliability in this measure, the more unreliable it is, the less robust the results can be; however the strong OR associated with the index test suggests that reliability is good enough to denote robustness of test).

My only real concern relates to the statistical approach. I would suggest that, in order to establish criterion validity, one would only need to focus on the index test, MRI, and consider important confounders in this very analysis. (The approach presented in the paper appears that there were candidate predictors searched for and tested, which is not exactly the same.)
The key challenge is that there were only 13 events and the OR is inflated therefore because of overfitting the model. I would use penalised logistic regression instead; I would then calibrate the OR using bootstrapping and come up with an optimism adjusted area-under-the-curve.
In revising this, it would be required to state more explicitly which covariates the final model included. (And how these were chosen).
An alternative, but less useful, approach is to demonstrate correlation between index and standard reference tests.

I think that the topic is timely and it would be excellent if the authors could employ advances statistical methods to deal with the problem of small event rate and risk of model overfitting. I would also suggest to distinguish clearer the issues of (1) establishing criterion validity of a novel index test, MRI and (2) identifying predictors for “AVN at 1 year as defined by Salter”. These are 2 different issues and should not be confused.

- Thank you for your valuable comments.

As the predictors for AVN in infants who underwent closed reduction and casting for treatment of DDH are not well-established, we explored possible predictors of AVN in this study. Therefore, the objective of this study is exploring predictors of AVN, including findings of contrast-enhanced MRI. We explored candidate predictors of AVN in the uni-variable analysis and the multi-variable model was constructed based on the clinical importance and findings from uni-variable analysis.

We used penalized logistic regression to reduce sparse data bias in the analysis, however, we made mistake while writing the manuscript and corrected accordingly. Also, we presented the optimism adjusted area-under-the-curve based on 1000 bootstrap samples for the multi-variable model as you recommended.
