# Supplementary material for: MRI risk factors for development of avascular necrosis after closed reduction of developmental dysplasia of the hip: Predictive value of contrast-enhanced MRI
Source: PLoS One. 2021 Mar 18;16(3):e0248701. doi: 10.1371/journal.pone.0248701 (PMC7971487; doi:10.1371/journal.pone.0248701)
Supplement: Attachment — Submitted filename: Response to reviewers-ver2.0.docx [file pone.0248701.s002.docx]

Response to Reviewers

**Reviewer #1**: The Authors have adequately addressed my critique and I am happy that the paper is ready for publication.

Thank you for your comments.

**Reviewer #2**:

1. Penalised logistic regression was used but there was no reference to the methods employed. Further, I was curious to learn that the SPSS statistical package can do this type of analysis; in my experience it can be done in SAS, STATA and R only (but I could be wrong). The same for the bootstrapping; also here there was no reference given about the methods used. I recommend adding references.

Thank you for your comments. We address the reference [22] (Firth D. Bias reduction of maximum likelihood estimates. Biometrika 1993; 80:27–38) and revised the statistical package that we used in this study (SAS)

2. Statistical section should include a clear description of how the modelling was done, i.e. what were candidate predictors (it seems that there is a 2nd section in the paper entitled “Multi variable analysis” but it would better be part of the section “Statistical analysis”.

Potential candidate risk factors for AVN considered in this study were clinical features including demographical characteristics and MRI features. According to your previous suggestion, we focused on risk factors analysis of index test, MRI.

3. It appears that most of the risk factors shown in table 2 were nonsignificant, yet they were subjected to a multivariable analysis – and remained nonsignificant. I don’t quite follow that approach (because the aim is not to develop a prediction rule but to identify independent risk factors).

In table 2, excessive abduction (p=0.026) and global decreased enhancement of the femoral head (p <0.001) showed statistically significant difference between AVN and non-AVN groups. Invisible secondary ossification center of the femoral head showed borderline significance (p=0.05). Containment of the femoral head has been considered as a risk factor to develop AVN, therefore, ‘eccentric reduction’ which was defined as a medial joint space widening more than 3 mm was included as a candidate predictor in multi-variable analysis.

There were four predictors in the penalised logistic regression model.

4. There were 13 AVN events (at hip level). There were five predictors included in the penalised logistic regression model, one of which was “eccentric reduction” but I could not find a definition of this variable.

It was an error to describe Table 3. There were four predictors in the penalised logistic regression model. Eccentric reduction was defined as > 3 mm of medial joint space distance.

5. It was a bit vague as to how these variables ended up in the final model. But what I understand was that there were several potential predictors, and 5 were selected for a multivariable model – and only 1 remained predictive of AVN. Unfortunately, this was the variable “global decreased enhancement of the proximal femoral epiphysis seen on MRI”. This, in turn, is not new knowledge or a surprise because this situation has long been known to indicate AVN.

There were still debate regarding predictive value of contrast enhancement of the femoral head in immediate post op. contrast enhanced MRI after closed reduction. Recently Nguyen et al. reported that neither of enhancement pattern nor percent enhancement predicted AVN on postoperative MRI (Nguyen, Jie C., et al. "Developmental dysplasia of the hip: can contrast-enhanced MRI predict the development of avascular necrosis following surgery?." *Skeletal Radiology* 50.2 (2021): 389-397)

6. Is your key message, therefore, is that no MRI-based predictors for AVN could be identified in this study - see table 3. It is already well-established that a lack of contract enhancement of the femoral head is a diagnostic MRI-criterion of AVN of the femoral head (at any age, also very young infants).

As I mentioned above, there were still debate regarding predictive value of contrast enhancement of the femoral head in immediate post op. contrast enhanced MRI after closed reduction. Geographic decreased enhancement with double rim sign is a well-known MRI features of AVN. But global decreased enhancement of the femoral head at immediate post op. MRI after closed reduction of DDH does not directly mean AVN. It is merely decreased contrast enhancement but it could be a risk factor of future AVN. Other MRI risk factors such as eccentric reduction, invisible secondary ossification center, and excessive abduction were not well-known MRI risk factors. Although both of eccentric reduction and invisible secondary ossification center were suggestive risk factors on X-ray and excessive abduction was a risk factor on clinical examination.

7. Was it not that your study merely confirmed this finding, but did show that other MRI criteria cannot predict X-ray-AVN? Therefore, the conclusion should reflect this. It looks to me that several MRI criteria were not helpful in predicting AVN, only one MRI criterion was.

Both of eccentric reduction and invisible secondary ossification center were suggestive risk factors on radiographic examination after closed reduction of DDH. Excessive abduction was also a well-known risk factor on clinical examination after closed reduction of DDH. In this study using MRI, contrast enhancement pattern could provide information regarding increased risk of future development of AVN.
